# Gastric Cancer Peritoneal Staging: Progress and Persistent Challenges

**DOI:** 10.1007/s12029-026-01404-2

**Published:** 2026-02-04

**Authors:** Eoghan Burke

**Affiliations:** The Royal College of Surgeons in Ireland, Dublin, Ireland

**Keywords:** Gastric cancer, Peritoneal staging, Cytology, Peritoneal cell-free DNA, Peritoneal lavage

## Abstract

Staging laparoscopy with peritoneal lavage has become a cornerstone of gastric cancer staging, particularly for identifying occult peritoneal disease. Recent Dutch and European Delphi consensus studies have now established broad agreement on indications for staging laparoscopy, systematic peritoneal inspection, and technical aspects of peritoneal lavage. However, while consensus on how to perform the procedure has advanced substantially, there remains striking heterogeneity in how peritoneal lavage cytology specimens are handled and interpreted by pathology laboratories. A recent multicenter Japanese survey demonstrated more than a tenfold variation in cytology positivity rates across high-volume centres, closely associated with differences in laboratory methodology. Similar variability has been acknowledged in European centres but remains largely unquantified. This diagnostic inconsistency has become increasingly consequential as novel treatment strategies for cytology-positive and low-volume peritoneal disease, such as Normothermic Intraperitoneal and Systemic chemotherapy (NIPS), cytoreductive surgery with HIPEC, and pressurised intraperitoneal aerosol chemotherapy (PIPAC), enter clinical practice. Failure to address cytological heterogeneity risks misclassification, inappropriate treatment allocation, and inequitable access to emerging therapies. Future efforts should focus on extending consensus into the pathology laboratory and exploring molecular adjuncts, including peritoneal cell-free DNA, to reduce diagnostic variability.

## Introduction

Peritoneal dissemination remains the most common and prognostically adverse metastatic pathway in gastric cancer. Conventional imaging techniques, including computed tomography and PET-CT, demonstrate limited sensitivity for detecting low-volume peritoneal disease, leading to the widespread adoption of staging laparoscopy with peritoneal lavage cytology as a key diagnostic step. Positive peritoneal cytology in the absence of macroscopic disease (P0/Cyt+) is classified as stage IV disease and is associated with poor survival [1, 2], yet may represent a biologically distinct subgroup increasingly targeted by evolving therapeutic strategies.

For many years, substantial variation existed in the indications for staging laparoscopy and in the technical execution of the procedure itself. Recently, however, this landscape has changed. National and international Delphi consensus initiatives have successfully harmonised surgical practice, defining when staging laparoscopy should be performed and how peritoneal inspection and lavage should be undertaken [3, 4]. In contrast, the cytological assessment of peritoneal lavage specimens has remained largely outside the scope of these initiatives.

As a result, gastric cancer staging now sits at an uncomfortable intersection: highly standardised surgical sampling paired with poorly standardised diagnostic interpretation. This imbalance has important clinical implications, particularly as treatment paradigms for peritoneal and cytology-positive disease rapidly evolve.

## Consensus Achieved: Staging Laparoscopy and Peritoneal Lavage

The Dutch nationwide Delphi consensus study published in 2024 represents a landmark effort to standardise staging laparoscopy in gastric cancer [3]. Consensus was reached on key aspects, including patient selection (cT3–4, cN+, diffuse-type tumours), systematic peritoneal inspection using the Peritoneal Cancer Index, and routine performance of peritoneal lavage or ascites aspiration for cytology. Importantly, the group aligned lavage volumes and sampling locations with AJCC staging recommendations [2, 3].

Building on this work, a large European Delphi study involving surgeons from 16 countries confirmed and expanded these findings [4]. This initiative established consensus on routine lavage during staging laparoscopy, recommended sampling of bilateral subphrenic spaces and the pouch of Douglas, and defined minimum instilled and aspirated volumes. Together, these studies represent a major step toward uniform surgical staging practice across Western gastric cancer centres.

However, both initiatives deliberately focused on surgical technique. Cytology was consistently mandated but not operationally defined. Neither study provided guidance on specimen handling, fixation, cytopreparation, staining, reporting thresholds, or quality assurance, elements that ultimately determine whether free cancer cells are detected.

### Persistent Diagnostic Heterogeneity: Lessons from Japan

The consequences of this omission are clearly illustrated by a recent multicenter Japanese survey published in *Annals of Surgery Open* [5]. In this study, 61 high-volume gastric cancer centres within the Japan Clinical Oncology Group completed a detailed questionnaire on cytological methodology, linked to aggregated cytology positivity rates from more than 11,000 patients with clinical T3–T4 disease.

Despite operating within a healthcare system where peritoneal cytology is deeply embedded in staging guidelines [1], the authors identified striking interinstitutional variability. Median cytology positivity was 8.5%, but rates ranged from 2.1% to 28.3% [5]. Differences in positivity were associated with laboratory-level practices, including lavage volume, use of additives, cytopreparation technique, fixation method, and adoption of rapid intraoperative cytology.

Notably, institutions with higher positivity rates more frequently employed ethanol fixation and smaller lavage volumes, suggesting that technical decisions at the laboratory bench may substantially influence staging outcomes. The implication is unsettling: a patient’s metastatic classification may depend as much on institutional cytology practice as on tumour biology.

## Europe: Variability Acknowledged but Unresolved

While no European study has yet replicated the methodological rigor of the Japanese survey, evidence of similar heterogeneity is emerging. As part of the European Delphi process, an inventory of cytological processing methods was conducted across steering committee centres and reported in supplementary material [6]. This inventory revealed wide variation in fixation methods, staining protocols, and reporting practices, even among expert institutions engaged in consensus development.

Importantly, this variability persists despite convergence on surgical technique and lavage sampling. Thus, Europe appears to have reached consensus on how to obtain peritoneal samples, while leaving unresolved the question of how those samples should be processed and interpreted. The absence of outcome-linked cytological standardisation raises concerns about interinstitutional comparability, stage migration, and the reproducibility of clinical research.

## Why this Matters Now: Therapeutic Consequences

Historically, positive peritoneal cytology was viewed primarily as a contraindication to curative surgery. Today, this paradigm is shifting. Increasingly, patients with P0/Cyt + disease or limited peritoneal involvement are being considered for aggressive multimodal strategies, including Normothermic Intraperitoneal and Systemic Chemotherapy (NIPS), cytoreductive surgery with HIPEC, and Pressurised Intraperitoneal Aerosolised Chemotherapy (PIPAC) [7–9].

In this evolving landscape, diagnostic accuracy is no longer academic. Variability in cytological detection undermines the validity of clinical trials and complicates comparisons of outcomes across institutions and countries.

As treatment pathways increasingly diverge based on cytology status, inconsistent diagnostic practices risk introducing inequity into patient selection and outcome reporting.

## Looking Forward: Beyond Conventional Cytology

The limitations of conventional cytology raise the question of whether standardisation alone is sufficient. Even with harmonised protocols, cytology remains observer-dependent and technically sensitive. Molecular approaches, particularly analysis of peritoneal cell-free DNA (cfDNA), offer a potential complementary strategy.

Emerging evidence indicates that tumour-derived DNA can be detected in peritoneal fluid and may improve sensitivity for identifying minimal residual peritoneal disease compared with cytology alone [10]. Early translational studies suggest that peritoneal cfDNA correlates with tumour burden and prognosis and may identify disease missed by cytology, particularly in low-volume or occult settings.

While peritoneal cfDNA remains investigational and requires prospective validation, it offers several theoretical advantages: quantitative assessment, reduced observer dependence, and improved reproducibility across centres. Importantly, the recent achievement of surgical consensus provides a unique platform for evaluating such molecular adjuncts in a standardised sampling environment.

## Conclusions and Call To Action

The gastric cancer community has made substantial progress in standardising staging, laparoscopy, and peritoneal lavage. Yet, this progress has exposed a critical weakness: persistent and clinically consequential heterogeneity in cytological assessment. As therapeutic options for peritoneal and cytology-positive disease expand, the cost of diagnostic inconsistency continues to rise.

Future consensus efforts must extend beyond the operating theatre and into the pathology laboratory. Parallel investment in cytological standardisation, quality assurance, and molecular adjuncts such as peritoneal cfDNA is urgently needed. Without this, the promise of precision staging risks being undermined by diagnostic uncertainty.

Table 1 despite recent consensus on the indications and technical performance of staging laparoscopy and peritoneal lavage, substantial variability persists in the cytological assessment of peritoneal lavage specimens. This table summarizes major domains in which interinstitutional differences occur, with illustrative examples and their potential clinical consequences. Variability in cytological collection, processing, interpretation, and reporting may influence cytology positivity rates, disease staging, and subsequent treatment allocation, particularly in the context of emerging therapeutic strategies for cytology-positive and low-volume peritoneal disease.

**Table 1:** Key sources of variability in peritoneal lavage cytology for gastric cancer and their potential clinical implications

Domain	Examples of variability	Potential clinical implications
Sample collection	Lavage volume; number and location of sampling sites	Dilution of malignant cells; reduced sensitivity
Specimen handling	Time to fixation; use of additives	Cellular degradation; false-negative results
Cytopreparation	Direct smears vs centrifugation; scraping techniques	Variable cell yield and morphology
Fixation and staining	Ethanol vs air-dried fixation; Pap vs combined stains	Differences in diagnostic clarity and positivity rates
Diagnostic workflow	Rapid intraoperative vs routine cytology	Trade-off between speed and diagnostic accuracy
Reporting standards	Binary vs equivocal categories; institutional thresholds	Stage migration; inconsistent treatment allocation

## Data Availability

No datasets were generated or analysed during the current study.
